# Small RNAs as therapeutic agents: From catalytic motifs to regulatory pathways

**DOI:** 10.1016/j.ymthe.2025.03.053

**Published:** 2025-04-02

**Authors:** John J. Rossi, Saumya Das

**Affiliations:** 1 Center for RNA Biology and Therapeutics, Beckman Research Institute of City of Hope, Duarte, CA, USA; 2 Harvard Medical School, Cardiovascular Research Center, Massachusetts General Hospital, Boston, MA, USA

**Keywords:** ribozymes, small interfering RNAs, siRNAs, transfer RNA-derived small RNAs, tDRs, antisense oligonucleotides, ASOs, small nuclear-targeting RNAs

## Abstract

RNA molecules have long been recognized for their central role in protein synthesis, primarily as messengers (mRNAs), ribosomal components, and adaptors (transfer RNAs). Over the past few decades, however, the discovery of small RNAs with regulatory or catalytic functions has dramatically expanded our understanding of RNA biology. These small RNAs can target specific transcripts for cleavage, alter mRNA translation, direct epigenetic changes at gene promoters, or even guide enzyme complexes to their substrates. In this review, we highlight and discuss the therapeutic potential of key classes of small RNAs, including ribozymes, RNA interference elements, antisense oligonucleotides, small nuclear-targeting RNAs, and transfer RNA-derived small RNAs.

## Graphical abstract

**Figure:**
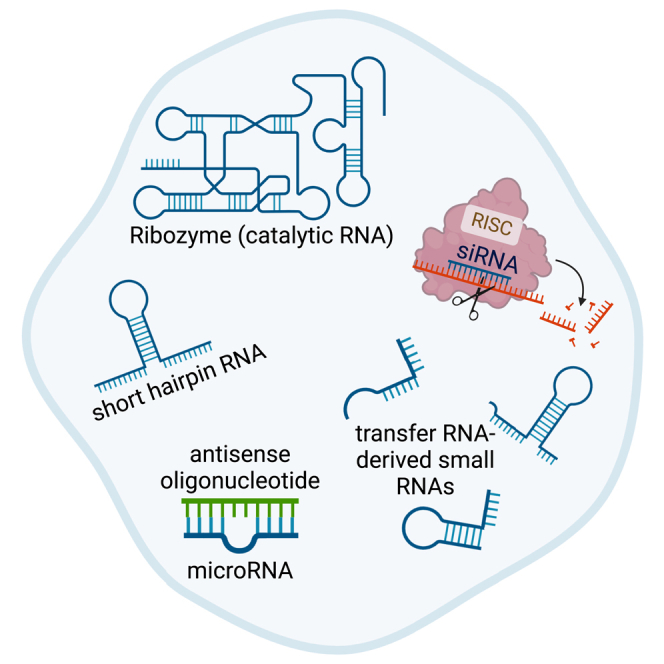

## Teaser

Small RNAs have been seen in cells for several decades but only recently have they been recognized for their therapeutic potential. This article summarizes the history and development of small RNAs as therapeutic agents.

## Introduction

RNAs can have coding, structural, or regulatory roles. Within the past 2 decades, it has become widely recognized that small RNAs play important roles in controlling gene expression. This article highlights the primary classes of small RNAs that have roles in regulating gene expression through their interactions with messenger RNA (mRNA) and discuss their therapeutic potential.

## Discovery and development of catalytic RNAs (ribozymes)

One of the earliest indications that RNA could harbor enzymatic activity emerged from studies of certain plant pathogenic RNAs called viroids and virusoids. These circular RNAs undergo self-cleavage via a structured catalytic element known as the hammerhead ribozyme.^1^^,^^2^ Forster and Symons subsequently demonstrated that the self-cleaving motif within the hammerhead ribozyme could be harnessed to act in *trans*, functioning independently of the larger viroid RNA to cleave target RNAs.

**Figure 1:**
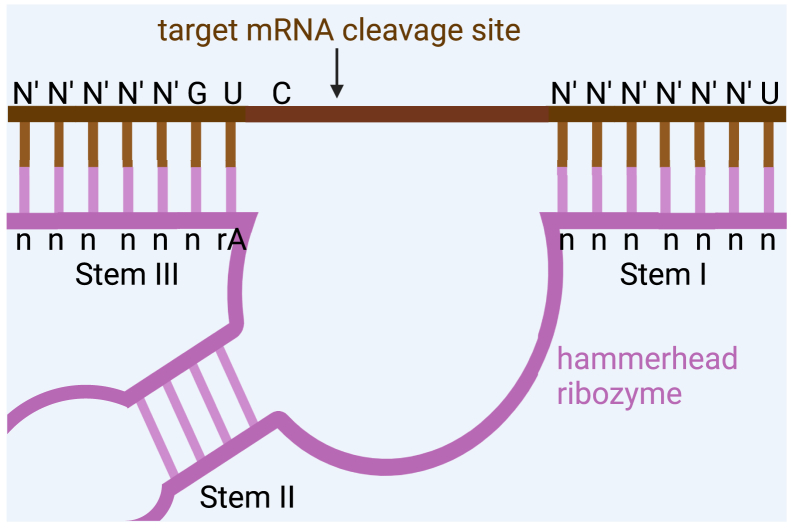
Schematic of the hammerhead ribozyme catalytic center The ribozyme core is flanked by Watson-Crick base pairing arms. The target RNA strand contributes a GUC triplet that forms a critical part of the catalytic pocket.^3^ Created in BioRender, https://BioRender.com/umx486t.

Structurally, the hammerhead ribozyme contains a small catalytic core flanked by arms of single-stranded RNA that can be designed to base pair with complementary sequences in the target transcript (Figure 1). Critically, the substrate itself must harbor a GUC triplet at the cleavage site; hybridization of this triplet to the catalytic core of the ribozyme is essential for efficient catalysis.^1^^,^^2^

Because of their well-defined structure and relatively small size, hammerhead ribozymes were among the first RNA-based motifs investigated for therapeutic applications aimed at cleaving pathogenic mRNAs. Two other self-cleaving motifs, the hairpin ribozyme, derived from the tobacco ringspot virus,^4^ and the hepatitis delta virus (HDV) ribozyme,^5^ were similarly demonstrated to have the potential to destroy mRNA targets in a sequence-specific manner.

## Early *in vitro* and cellular testing of ribozymes

Initial *in vitro* studies on ribozymes explored how various design features, such as flanking-arm length and target RNA secondary structure, influence catalytic efficiency. These experiments also highlighted the importance of divalent cations (e.g., Mg^2+^) for optimal ribozyme activity. Nonetheless, ribozyme performance in live cells often deviated from *in vitro* predictions, owing to the complexity of intracellular environments, which include RNA-binding proteins that can alter ribozyme folding or substrate accessibility.

A critical breakthrough in cell-based studies came when Sarver et al. demonstrated that hammerhead ribozymes could block HIV replication in T lymphocytes.^6^ Rather than delivering chemically synthesized ribozymes, the investigators used a gene-therapy approach, embedding the ribozyme within an expression cassette that could be transcribed in the cell. This strategy effectively merged the burgeoning field of gene therapy with ribozyme technology.

## Additional catalytic RNA motifs

Beyond the hammerhead, hairpin, and HDV ribozymes, other catalytic RNAs hold therapeutic promise. For example, the RNA component of the essential endonuclease RNase P can be engineered to guide this enzyme to cleave target transcripts in *trans*, although the guide RNA itself is not catalytic.^7^ Similarly, despite being comparatively large, the first catalytic RNA discovered—the group I intron from *Tetrahymena*—can be re-engineered to cleave disease-relevant RNAs.^8^ Collectively, these catalytic RNAs underscore the wide-ranging possibilities for therapeutic transcript ablation via RNA-based enzymes.

## RNA interference and microRNAs

**Figure 2:**
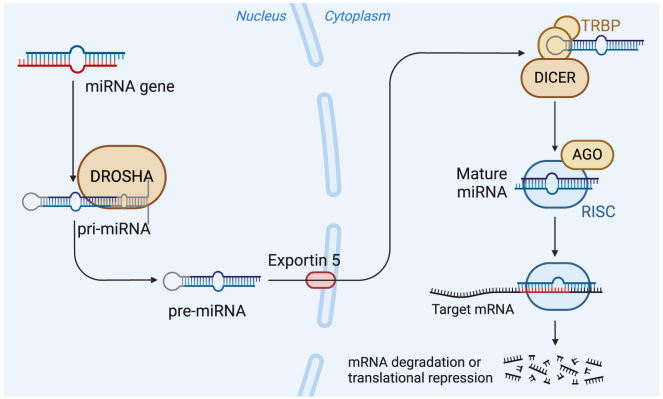
Overview of RNA interference Mammalian primary microRNA transcripts (pri-miRNA) are transcribed in the nucleus and cleaved by the microprocessor complex (drosha) to produce (∼30 bp) short hairpin RNAs (shRNAs) called pre-miRNA. Exportin 5 transports the pre-miRNA to the cytoplasm where it binds with Dicer and TAR RNA-binding protein (TRBP). Dicer cleaves the terminal loop of pre-miRNA and induces formation of an RNA-induced silencing complex (RISC)-loading complex with an Argonaute (AGO) protein. The mature RISC can regulate gene expression by promoting mRNA degradation and directing transcriptional gene silencing of the target gene loci. Created in BioRender, https://BioRender.com/liu2f2j; adapted from Team, B. (2021) microRNA in Cancer, https://app.biorender.com/biorender-templates/figures/all/t-60087a1abb5c0200a2b0d483-microrna-in-cancer.

One of the most significant advances in small RNA biology is the discovery of RNA interference, wherein short double-stranded RNAs (dsRNAs) trigger the degradation or translational repression of complementary mRNAs.^9^ In eukaryotic cells, long dsRNAs are processed by the enzyme Dicer into 21- to 25-nt small interfering RNAs (siRNAs). These siRNAs associate with the RNA-induced silencing complex (RISC) (Figure 2), which contains an Argonaute family protein that catalyzes cleavage of the target mRNA (previously reviewed by Setten et al.^10^).

Therapeutically, siRNAs can be designed to silence virtually any desired gene with remarkable specificity. However, potential off-target effects, often arising when siRNAs behave like microRNAs (miRNAs), remain a major concern. In fact, the notion that endogenous short hairpin RNAs give rise to miRNAs was itself a turning point in understanding gene regulation.^11^ These miRNAs typically bind Argonaute proteins and pair partially with mRNA targets, often via a 6- to 8-nt “seed” region, leading to translational repression or destabilization of hundreds of potential targets.

Given their broad regulatory power, miRNAs are now implicated in almost every aspect of cell physiology and are attractive therapeutic targets. Although to date there are no known miRNA therapeutics, it is conceivable that strategies such as delivering miRNA mimics to restore beneficial miRNA function or using antisense oligonucleotides (ASOs, sometimes called antagomirs) to inhibit pathogenic miRNAs could eventually become approved therapeutics. Balancing potency and specificity and minimizing off-target or immunogenic effects have become a critical focus in miRNA-based drug development.

## ASOs

ASOs are short, synthetic nucleic acids designed to bind complementary sequences in target RNAs. By virtue of this base pairing, ASOs can modulate RNA function in several ways: they can induce RNA cleavage by endogenous RNase H, block translation machinery, influence splicing patterns, or otherwise disrupt normal RNA-protein interactions. Over the past 3 decades, ASOs have moved from experimental gene-silencing tools to bona fide therapeutic agents, with multiple US Food and Drug Administration (FDA)-approved drugs on the market.^12^

Paul Zamecnik is widely regarded as the first to propose that synthetic oligonucleotides could be harnessed to block protein translation for therapeutic purposes. In his groundbreaking work, he showed that binding of a custom-designed oligodeoxynucleotide to Rous sarcoma virus RNA inhibited viral RNA translation and, consequently, halted viral replication.^13^^,^^14^ Although this method of translational inhibition via steric hindrance remains a viable therapeutic strategy, modern ASOs instead rely on RNase H1, an endonuclease present in most cells that promotes the cleavage of RNA in an RNA-DNA heteroduplex.^15^ RNase H1 degradation relies on 5–10 DNA nucleotides being incorporated into the ASO. This is often accomplished through a chimeric “gapmer” design, wherein a central block, or gap, of DNA nucleotides is flanked on both ends by chemically modified RNA-like nucleotides, which allows RNase H-mediated binding while increasing binding affinity and protecting the ASO from nuclease-mediated breakdown.

Another specialized application of ASOs is splicing modulation through exon skipping, wherein ASOs target pre-mRNA splice sites or splicing enhancer motifs, prompting the cellular machinery to skip a particular exon. By excluding an exon that contains or flanks a pathogenic mutation, these ASOs can effectively reframe a mutant transcript and restore partial protein function. This approach has been prominently demonstrated in Duchenne muscular dystrophy (DMD). In DMD, exon-skipping ASOs enable the production of a truncated yet functional dystrophin protein, which alleviates disease severity. This strategy has already led to multiple FDA-approved therapies (Table 1)^16^^,^^17^^,^^18^^,^^19^ for distinct DMD mutations, illustrating the power of precise splicing modulation to combat inherited disorders. As research continues to advance, exon skipping holds promise for numerous additional genetic diseases involving splicing abnormalities or protein misfolding.^20^

**Table 1:** Overview of the currently FDA-approved small RNA therapies, highlighting the class of small RNA, year of approval, clinical indications, and mechanism of action

Drug Name	Class	Approval year	Indication	Mechanism of action
Fomivirsen (Vitravene)	ASO	1998	CMV retinitis	binds to CMV mRNA, blocking viral protein synthesis
Mipomersen (Kynamro)	ASO	2013	homozygous familial hypercholesterolemia	induces RNAse H-mediated degradation of ApoB-100 mRNA, reducing LDL-cholesterol production
Eteplirsen (Exondys 51)	ASO	2016	DMD amenable to exon 51 skipping	induces skipping of exon 51 in the dystrophin gene
Nusinersen (Spinraza)	ASO	2016	SMA	binds to SMN2 pre-mRNA, enhancing inclusion of exon 7 and increasing functional SMN protein
Inotersen (Tegsedi)	ASO	2018	hATTR	induces RNAse H-mediated degradation of TTR mRNA, reducing toxic TTR protein
Patisiran (Onpattro)	siRNA	2018	hATTR	silences TTR mRNA via RISC
Golodirsen (Vyondys 53)	ASO	2019	DMD amenable to exon 53 skipping	induces skipping of exon 53 in the dystrophin gene
Givosiran (Givlaari)	siRNA	2019	AHP	silences ALAS1 in liver, reducing neurotoxic precursors
Viltolarsen (Viltepso)	ASO	2020	DMD amenable to exon 53 skipping	induces skipping of exon 53 in the dystrophin gene
Lumasiran (Oxlumo)	siRNA	2020	PH1	silences the HAO1 gene (glycolate oxidase), reducing oxalate accumulation
Casimersen (Amondys 45)	ASO	2021	DMD amenable to exon 45 skipping	induces skipping of exon 45 in the dystrophin gene
Inclisiran (Leqvio)	siRNA	2021	hypercholesterolemia and mixed dyslipidemia	targets PCSK9 mRNA to lower LDL cholesterol
Vutrisiran (Amvuttra)	siRNA	2022	hATTR	silences TTR mRNA via RISC
Tofersen (Qalsody)	ASO	2023	ALS	reduces SOD1 mRNA, lowering SOD1 protein

AHP, acute hepatic porphyria; ALAS1, amino levulinate synthase 1; ALS, amyotrophic lateral sclerosis; CMV, cytomegalovirus; hATTR, hereditary transthyretin-mediated amyloidosis; LDL, low-density lipoprotein; PH1, primary hyperoxaluria type 1; SMA, spinal muscular atrophy; SOD1, superoxide dismutase 1; TTR, transthyretin.

## Small nuclear-targeting RNAs for gene activation or silencing

An emerging area of small RNA research is determining how short dsRNAs can enter the nucleus and modulate gene transcription.^21^^,^^22^ These small nuclear-targeting RNAs, which can act as gene-activating or gene-silencing RNAs, are thought to recruit chromatin-remodeling complexes or transcription factors to specific gene promoters. In some cases, they induce gene activation by promoting a more open chromatin conformation; in others, they induce transcriptional gene silencing by triggering heterochromatin formation. Although the precise mechanisms remain incompletely defined, these findings broaden the scope of RNA-based regulation to the level of epigenetics.^23^

## tRNA-derived small RNAs

Transfer RNAs (tRNAs) are key adaptor molecules in translation.^24^ Accumulating evidence shows tRNAs can be cleaved into smaller fragments^25^: cleavage in the anti-codon loop yields tRNA halves or stress-induced tiRNAs,^26^^,^^27^ while cleavage in the T- or D-arm yields smaller tRNA-derived fragments (tRFs).^28^^,^^29^ Referred to in the literature variously as tRFs, tiRNAs, tsRNAs, or tDRs, we refer to these RNAs as tRNA-derived small RNAs (tDRs) based on proposed standardized nomenclature.^30^ The processing of mature tRNAs into tDRs appears to be mediated by ribonucleases such as Dicer, ELAC2, and Angiogenin and is important for gene regulation and cellular response to stress.^26^^,^^28^^,^^29^^,^^31^^,^^32^^,^^33^ Although recognized only recently, tDR biogenesis has been described even in bacteria,^34^ suggesting an important evolutionary conserved role.^25^ Indeed, studies over the past decade illuminate their role in diverse regulatory functions: they can silence RNA, modulate translation, promote stress granule formation, and even influence epigenetic inheritance.^26^^,^^28^^,^^29^^,^^35^^,^^36^ Dysregulation of tDRs has been documented in various diseases, including neurological disorders and cancer,^37^^,^^38^^,^^39^ raising the possibility of targeting them therapeutically.

## Therapeutic targeting of tDRs

Given their newly appreciated roles, tDRs have become targets for potential therapeutic intervention. In certain cancer models, ASOs containing locked nucleic acids (LNAs) have been designed to either antagonize or mimic dysregulated tDRs.^35^^,^^38^ Early studies did not rigorously assess whether parent tRNA molecules or isodecoders, tRNA molecules that share the same anticodon but have different sequences, were inadvertently targeted, which could lead to off-target effects. However, subsequent work using more refined LNA designs showed that it is possible to specifically deplete or restore tDR levels without significant effects on full-length tRNAs.^35^

*In vivo* approaches such as intraperitoneal injection of LNA gapmers or antagomirs can reduce tDR expression in tumor xenograft models and slow tumor growth.^35^ Conversely, synthetic “agomiRs” (dsRNAs initially developed to mimic miRNAs) have been used to increase expression of specific tDRs. Although details regarding how synthetic tDRs are processed intracellularly remain limited, some studies suggest they can modulate tumor biology by regulating the stability of mRNAs important for tumor growth and metastasis.^38^

These encouraging results highlight the therapeutic promise of tDR modulation. However, many questions remain about the optimal chemical modifications to ensure tDR stability and specificity, as well as the optimal platforms (e.g., lipid nanoparticles, polymer conjugates) for delivering tDRs to tissues of interest.^40^ Notably, while the aforementioned studies have used systemic (e.g., intraperitoneal or direct tumor injection) for targeting tDRs, there remains a significant gap in the knowledge about tissue distribution, off-target effects (including on other RNAs with sequence similarity to the targeted tDR), and relative efficacy/duration of effect of these molecules. As research into tDR biology advances, more precise strategies for harnessing or inhibiting these small RNAs in disease contexts are likely to emerge.

## FDA-approved small RNA therapeutics

In the last decade, we have witnessed an increasing pace of FDA-approved small RNA therapeutics, as they have evolved from an experimental concept to a transformative new class of drugs. The majority of FDA-approved drugs comprise ASOs and siRNAs targeting chronic inherited diseases caused by mutations in single genes, such as DMD and spinal muscular atrophy (SMA). Table 1 highlights FDA-approved ASOs and siRNAs and illustrates the increasing pace of approvals for small RNA therapeutics.

Collectively, these FDA-approved drugs illustrate the growing versatility and reliability of small RNA therapeutics. Chemical modifications (e.g., phosphorothioate backbones, 2′-*O*-modifications, LNAs), advanced conjugation strategies (e.g., GalNAc for hepatocyte targeting), and improved formulations (e.g., lipid nanoparticles) have overcome early challenges related to stability, delivery, and off-target effects. As a result, RNA-based interventions can treat not only rare and orphan genetic disorders but also more common metabolic and cardiovascular conditions.

Looking ahead, the pipeline of next-generation small RNA drugs continues to expand into oncology, infectious diseases, and additional neurological disorders. With each new FDA-approved drug, confidence in the transformative power of RNA therapeutics grows, setting the stage for a future in which precisely tailored oligonucleotides become routine clinical options for an ever-wider range of diseases.

## Concluding remarks

Small RNAs have revolutionized our understanding of gene regulation and opened new avenues for therapeutic intervention. From the early discovery of hammerhead ribozymes to the vast regulatory networks governed by siRNAs, miRNAs, small nuclear-targeting RNAs, and tDRs, it is evident that RNA molecules can function as catalysts, silencers, activators, or even epigenetic regulators. Each class of small RNA presents unique challenges related to delivery, specificity, and cellular context, but the overarching promise is substantial. As fundamental research continues to reveal the structural and functional nuances of these RNA species, clinical applications, ranging from antiviral therapies to anticancer regimens, are poised to become an integral part of precision medicine.

It has been only a few decades since the first small RNAs were described as potential therapeutics, yet discoveries continue to emerge, leading to disease-curing developments and numerous clinical trials featuring several FDA-approved small RNA drugs. Because most small RNA therapeutics rely on Watson-Crick base pairing to target RNA and DNA sequences, drug development can be both rapid and efficient. We have only begun to witness the full potential of small RNA therapeutics.

## Acknowledgments

J.J.R. and S.D. are cofounders of Switch Therapeutics, an siRNA company. S.D. is also a cofounder of Thryv Therapeutics, a precision medicine company, for which he holds equity and provides consulting. This work was supported by funding from the Lidow family foundation to J.J.R.; a research grant from Bristol Myers Squib to S.D.; and funding from the 10.13039/100000050National Heart, Lung, and Blood Institute (grant no. R35 HL 150807) to S.D. We thank Daniel J. Rossi for contributing content and Dr. Sarah T. Wilkinson for scientific editing support. The graphical abstract was created using BioRender (https://BioRender.com/w16h597).

## Author contributions

J.J.R. conceptualized, wrote, and revised this review. S.D. wrote the section on tRNA fragments.

## Declaration of interests

The authors declare no competing interests.
